# Supplementary material for: Two-Stage Evolution of Gamma-Phase Spherulites of Poly (Vinylidene Fluoride) Induced by Alkylammonium Salt
Source: Polymers (Basel). 2022 Sep 18;14(18):3901. doi: 10.3390/polym14183901 (PMC9504496; doi:10.3390/polym14183901)
Supplement: Supplementary file 1 [file polymers-14-03901-s001.zip › polymers-1891634-supplementary.pdf]

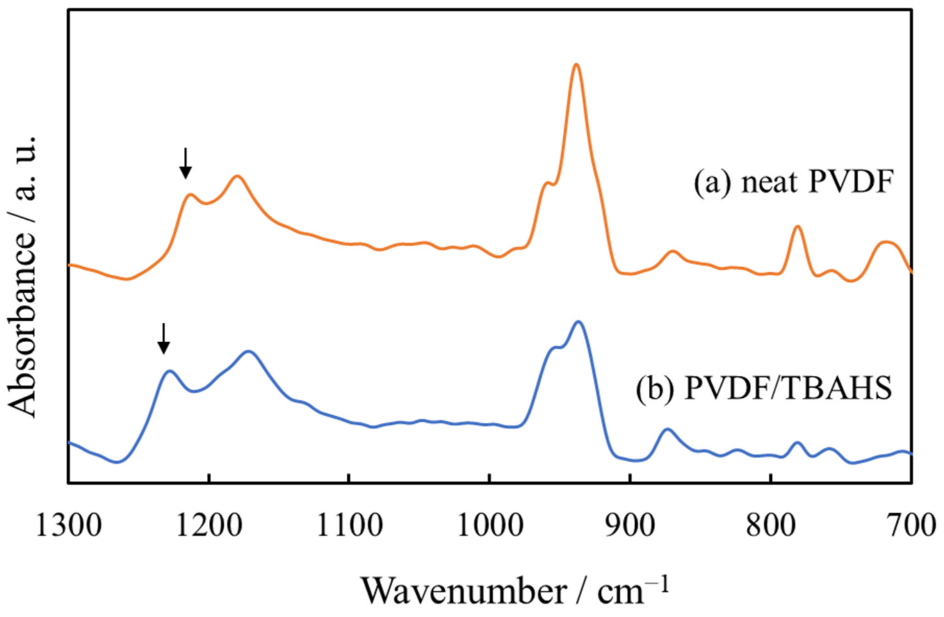

Figure S1. FT-IR spectra during isothermal crystallization at 165 °C: (a) α-phase spherulite of neat PVDF for 100 min, (b) γ-phase spherulite of PVDF/TBAHS for 40 min.
